# Surface-exposed loops L7 and L8 of *Haemophilus* (*Glaesserella*) *parasuis* OmpP2 contribute to the expression of proinflammatory cytokines in porcine alveolar macrophages

**DOI:** 10.1186/s13567-019-0721-4

**Published:** 2019-11-29

**Authors:** Ye Zhou, Saixiang Feng, Xinyi He, Qun Zhou, Yuanwei Wang, Hua Yue, Cheng Tang, Bin Zhang

**Affiliations:** 1 College of Life Science and Technology, Southwest Minzu University, Chengdu, 610041 China; 2 Key Laboratory of Ministry of Education and Sichuan Province for Qinghai-Tibetan Plateau Animal Genetic Resource Reservation and Utilization, Chengdu, 610041 China; 3 College of Veterinary Medicine, South China Agricultural University, Guangzhou, 510642 China

## Abstract

Outer membrane protein P2 (OmpP2) of the virulent *Haemophilus* (*Glaesserella*) *parasuis* has been shown to induce the release of proinflammatory cytokines. The OmpP2 protein is composed of eight or nine surface-exposed loops, but it is unclear which of them participates in the OmpP2-induced inflammatory response. In this study, we synthesized linear peptides corresponding to surface-exposed loops L1–L8 of OmpP2 from the virulent *H. parasuis* SC096 strain to stimulate porcine alveolar macrophages (PAMs) in vitro. We found that both L7 and L8 significantly upregulated the mRNA expression of interleukin (*IL*)-*1α*, *IL*-*1β*, *IL*-*6*, *IL*-*8*, *IL*-*17*, and *IL*-*23* and the chemokines *CCL*-*4* and *CCL*-*5* in a time- and dose-dependent manner. Additionally, we constructed *ompP2ΔLoop7* and *ompP2ΔLoop8* mutant SC096 strains and extracted their native OmpP2 proteins to stimulate PAMs. These mutant proteins induced significantly less mRNA expression of inflammatory cytokines than SC096 OmpP2. Next, the amino acid sequences of L7 and L8 from 15 serovars of *H. parasuis* OmpP2 were aligned. These sequences were relatively conserved among the most virulent reference strains, suggesting that L7 and L8 are the most active peptides of the OmpP2 protein. Furthermore, L7 and L8 significantly upregulated the NF-κB and AP-1 activity levels based on luciferase reporter assays in a dose-dependent manner. Therefore, our results demonstrated that both surface-exposed loops L7 and L8 of *H. parasuis* OmpP2 induced the expression of proinflammatory cytokines possibly by activating the NF-κB and MAPK signalling pathways in cells infected by *H. parasuis*.

## Introduction

*Haemophilus* (*Glaesserella*) *parasuis*, a member of the *Pasteurellaceae* family, is a common commensal extracellular bacterium that colonizes the upper respiratory tract in swine. Under certain circumstances, virulent strains of *H. parasuis* can cause Glässer’s disease, which is characterized by fibrinous polyserositis, polyarthritis, and meningitis [1]. *H. parasuis* infection causes a significant increase in mortality and morbidity in swine, leading to major economic losses in the pig industry. To date, 15 *H. parasuis* serovars with apparent differences in virulence have been described, and it is generally acceptable to use serovars as markers of pathogenicity [2]. *H. parasuis* infection induces a strong inflammatory response mainly mediated by cytokines and chemokines, including interleukin (*IL*)-*1α*, *IL*-*1β*, *IL*-*6*, *IL*-*8*, C–C motif chemokine ligand 4 (CCL-4, also known as macrophage inflammatory protein-1b, MIP-1b) and CCL-5 [3–6].

Outer membrane protein P2 (OmpP2), a member of the porin family, is the most abundant protein in the outer membrane of *H. parasuis* [7]. As a multifunctional protein, OmpP2 not only plays a major role in maintaining the structural membrane integrity and permeability [8] but also is believed to interact with host cells and tissues in regards to the pathogenesis and immunity to infections by gram-negative bacteria [9–11]. *H. parasuis* OmpP2 induces inflammatory responses and upregulates the expression of the proinflammatory cytokines *IL*-*1α*, *IL*-*1β*, *IL*-*6* and *IL*-*8* in porcine alveolar macrophages (PAMs) [12]. OmpP2 has two distinct structures and is composed of eight or nine surface-exposed loop regions with high sequence variability among serovars [13–15]. In most virulence serovar strains, two discontinuous sequence deletions of the short *ompP2* gene resulted in fewer predicted surface-exposed loops in the protein structure. In our previous study, we found that the reduced predicted surface-exposed loops in the *H. parasuis* OmpP2 protein may contribute to serum resistance and cytotoxicity to Marc-145 cells, suggesting that specific structural characteristics of the OmpP2 protein are involved in *H. parasuis* virulence [15, 16]. Nevertheless, it is uncertain whether these surface-exposed loops participate in proinflammatory responses.

In gram-negative bacteria, the ability to cause infections is partly attributed to the antigenic variability in all surface-exposed loops of OMPs, and these loops also play a key role in regulating both innate and adaptive immune responses [9, 17, 18]. In *Haemophilus influenzae*, synthetic peptides corresponding to loops L5, L6 and L7 of OmpP2 activated the JNK and p38 MAPK pathways and upregulated the IL-6 and TNF-α expression in host cells, with L7 being the most active peptide [17]. In *Klebsiella pneumonia*, loop L7 of porin OMPK36 is involved in the interaction with C1q, the first component of the classical pathway of the complement system, which is comparable to the C1q-LPS interaction [18]. However, it is unknown which of the predicted surface-exposed loops of *H. parasuis* OmpP2 plays an important role in inflammatory response. In this study, we used well-characterized PAMs to investigate the roles of loops L1–L8 of *H. parasuis* SC096 OmpP2 in the inflammatory response.

## Materials and methods

### Bacterial strains, plasmids, cells and culture conditions

The bacterial strains and plasmids used in this study are listed in Table 1. *Escherichia coli* plasmids were propagated in *E. coli* DH5α and grown in Luria–Bertani medium. *H. parasuis* strains were cultivated in Trypticase Soy Agar (TSA) and Trypticase Soy Broth (TSB) (Hopebio, Qinghai, China) supplemented with 10 µg/mL nicotinamide adenine dinucleotide (NAD) (Sigma-Aldrich, St Louis, MO, USA) and 5% (v/v) inactivated bovine serum at 37 °C in a 5% CO_2_-enriched atmosphere for 36 h. When required, the media were supplemented with kanamycin (30 µg/mL) or gentamicin (20 µg/mL). PAMs (3D4/2, ATCC, USA) were cultured in RPMI 1640 medium (Invitrogen, Carlsbad, CA, USA) containing 10% (v/v) foetal bovine serum. All cell culture media and supplements were purchased from Invitrogen Laboratories, Inc (Invitrogen, Carlsbad, CA, USA).

**Table 1:** **Strains and plasmids used in this study**

Strains or plasmids	Description	References
Strains		
*Escherichia coli* DH5α	FL80DlacZDM15D (lacZYA-argF) U169 recA1 endA1 hsdR17	Laboratory collection
*Haemophilus parasuis*		
SC096	Serovar 4 clinical isolate	[16]
*ΔompP2*	SC096 *ΔompP2*, Gm^R^	
*ompP2*Δ*Loop7*	SC096 *ompP2*Δ*Loop7*, Km^R^	This study
*ompP2*Δ*Loop8*	SC096 *ompP2*Δ*Loop8*, Km^R^	
Plasmids		
pMD-19T	T-vector, Amp^R^	Takara
pK18mobsacB	Suicide and narrow-broad-host vector, Kan^R^	[33]
pBAD18-Km	Kan resistance cassette-carrying complement vector, Kan^R^	[34]
ZY01	A fragment containing the upstream and downstream sequences of the OmpP2 gene in pK18mobsacB, Kan^R^	This study
pZY02	A fragment containing the upstream and downstream sequences of the Loop7 gene in pK18mobsacB, Kan^R^	This study
pZY03	A fragment containing the upstream and downstream sequences of the Loop8 gene in pK18mobsacB, Kan^R^	This study

### Preparation of native OmpP2 proteins and synthesis of linear peptides corresponding to loops L1–L8

Native OmpP2 proteins were purified from the *H. parasuis* SC096 strain as previously described [19]. The purified OmpP2 protein was suspended in 10 mM HEPES buffer (pH 7.4) containing 0.25% (w/v) sodium lauryl sulfate (SLS) and was extensively dialyzed and analysed by 12% SDS-PAGE. Polymyxin B (30 µg/mL, Biosharp, Hefei, Anhui, China) was mixed with porins at 37 °C for 24 h to neutralize the biological activity of possible traces of lipopolysaccharide (LPS) that were revealed on SDS-PAGE gels stained with silver nitrate and by the Limulus amoebocyte lysate assay. Finally, the purified porins were identified by the matrix-assisted laser desorption/ionization (MALDI)-time-of-flight (TOF)-TOF method.

The surface-exposed loops of OmpP2 from the *H. parasuis* SC096 strain were predicted using PRED-TMBB software. The linear peptides corresponding to loops L1–L8 were synthesized by Sangon Biotech Co., Ltd. (Shanghai, China). Amino acid sequences of all synthesized peptides are reported in Additional file 1. The scrambled peptide had the same amino acid composition as loop L7, but in a different order. Sequence quality and quantity were determined by high-performance liquid chromatography (HPLC) and mass spectrometry analyses. All purified peptides were obtained with good yields and purity > 95%.

### Cell viability

The viability of PAMs was measured by the cell counting kit-8 (CCK-8) assay (MCE, USA). Briefly, PAMs were seeded into 96-well plates at 1 × 10^5^ cells/well and then stimulated with OmpP2 (5 µg/mL and 10 µg/mL) or loops L1–L8 (130 nmol/mL and 260 nmol/mL) for 6 and 12 h at 37 °C under 5% CO_2_. CCK-8 solution (10 µL) was added to each well and incubated for 90 min at 37 °C, and the optical density was then measured at 450 nm. Cell viability was calculated according to the following formula: cell viability (%) = (experimental well − blank well/control well − blank well) × 100. The data are expressed as the mean ± standard deviation (SD) of triplicate samples from at least three independent experiments.

### PAM stimulation with native OmpP2 protein or the linear peptide of loops L1–L8

PAMs were stimulated as previously described with some modifications. Briefly, 24-well tissue culture plates were seeded with 5 × 10^5^ cells in RPMI 1640 (Invitrogen) containing 10% heat-inactivated foetal bovine serum at 37 °C in a humidified incubator with 5% CO_2_ for 24 h. When cultures were semi-confluent, cells were stimulated in triplicate wells for 6 h and 12 h with OmpP2 (5 µg/mL and 10 µg/mL), synthesized linear peptides corresponding to loops L1–L8 of OmpP2 or scrambled peptide (130 nmol/mL and 260 nmol/mL). Cells were stimulated with HEPES buffer (pH 7.4) containing 0.25% (w/v) SLS as a mock control, and the untreated cells were negative control. All the above assays were performed in triplicate and replicated three times.

PAMs were lysed for total RNA purification, and cDNA was synthesized using the PrimeScript RT Reagent Kit with gDNA Eraser (TaKaRa, Dalian, China). The oligonucleotide primers used for PCR (Additional file 2) were synthesized by Sangon Biotech. Real-time PCR was performed with the primers using SYBR Premix Ex TaqTM II (Tli RNaseH Plus; TaKaRa) using the QuantStudio 3 Real-Time PCR System (Thermo Fisher Scientific, USA) according to the manufacturers’ instructions. Based on a reported method [20], accurate normalization of the real-time PCR data was performed by geometric averaging of two internal control genes, glyceraldehyde-3-phosphate dehydrogenase (*GAPDH*) and ribosomal protein L-4 (*RPL4*). The data from at least three independent experiments were analysed using geometric averaging of two internal control genes in triplicate.

The concentrations of IL-6 and IL-8 secreted from PAMs stimulated with OmpP2, loops L7 and L8 or scrambled peptide in culture supernatants were measured using specific porcine ELISA kits (R&D Systems, MN, USA), following the manufacturer’s instructions. The optical density value of each well was determined using a microplate reader (Bio-Rad, Hercules, CA, USA) at 450 nm. To ensure the repeatability of the experiment, each sample was added to duplicate wells for each time point in each ELISA plate, and the average concentration was used as the protein level (pg/mL) in cell culture supernatants.

### Construction of plasmids and mutants

A 1.529-kb PCR fragment containing the 965-bp kanamycin resistance cassette amplified from the pBAD18-Km plasmid and 564 bp downstream from the TAA stop codon in the *ompP2* gene of strain SC096 was amplified using overlap extension PCR with primers P1 and P2 (Additional file 2). The fragment was subsequently cloned into the plasmid pk18mobsacB to create pZY01. Next, a 1.501-kb PCR fragment containing 589 bp upstream of the ATG start codon and 912 bp upstream of the loop L7 region of *ompP2* was amplified with primers P3 and P6, and a 176-bp PCR fragment containing the downstream region of loop L7 region of *ompP2* was amplified with primers P4 and P5. Both fragments were amplified using overlap extension PCR with primers P3 and P4 and subsequently cloned into plasmid pZY01 to create pZY02. Similarly, a 1.612-kb PCR fragment containing 589 bp upstream of the ATG start codon and 1.023 kb upstream of the loop L8 region of *ompP2* was amplified with primers P3 and P8, and a 72-bp PCR fragment containing the downstream region of loop L8 region of *ompP2* was amplified with primers P4 and P7. Both fragments were amplified using overlap extension PCR with primers P3 and P4 and subsequently cloned into plasmid pZY01 to create pZY03. All plasmids were transformed into *E. coli* DH5α by a CaCl_2_-mediated transformation method.

The natural transformation assay was performed according to Zhang et al. [16]. Briefly, the recipient SC096 *ΔompP2* mutant was cultured overnight at 37 °C and resuspended in TSB at 5 × 10^10^ colony-forming units/mL. A 20-μL aliquot of the suspension was spotted onto a TSA plate and spread on a small area. Next, 1 µg of donor DNA plasmid (pZY02 or pZY03 resuspended in TE buffer) was added, mixed and incubated for 5 h at 37 °C. Bacterial cells were scraped and inoculated in medium containing kanamycin and incubated at 37 °C for 2–3 days. Finally, bacterial cells were collected and plated on a TSA plate containing NAD, serum and kanamycin. For the negative control, TE buffer was added to a bacterial spot in place of the donor DNA.

When pZY02 and pZY03 used as donor DNA were introduced by natural transformation into the recipient SC096 *ΔompP2* mutant, many kanamycin-resistant transformants were obtained. Colony PCR was used to check some of the transformants, and the resulting mutants were designated as *ompP2ΔLoop7* and *ompP2ΔLoop8*, respectively.

### *H. parasuis* strains and sequences from databases

The OmpP2 protein sequences of 15 serovars from *H. parasuis* reference strains were obtained from NCBI. Comparisons of the amino acid sequences of loops L7 and L8 were performed using CLC Sequence Viewer 8.0 (CLC Bio-Qiagen, Aarhus, Denmark).

### Transfection and reporter assay

Transient transfection was performed using Lipofectamine 2000 (Invitrogen). PAMs were co-transfected with NF-κB or MAPK luciferase reporter plasmids, pNF-κb-Luc or pAP-1-Luc (Stratagene, USA), respectively, and the Renilla luciferase construct pRL-TK (Promega, Madison, WI, USA), which served as an internal control. At 24 h after transfection, PAMs were inoculated or control-inoculated with porins, loops L7 and L8 or scrambled peptide for 12 h. Cells were harvested, and luciferase activity was measured using a dual-luciferase assay system (Promega) according to the manufacturer’s instructions. Data represent relative firefly luciferase activity normalized to Renilla luciferase activity. All the above assays were performed in triplicate and replicated three times.

### Statistical analysis

Data were subjected to one-way analysis of variance (ANOVA) using the general linear model procedures of the IBM SPSS Statistics 26.0 statistical software program (Chicago, IL, USA). Duncan’s multiple comparison test was used to identify differences among group means. *p* < 0.05 was considered statistically significant.

## Results

### Extraction of OmpP2 from the *H. parasuis* SC096 strain

Using our reported method [19], the native OmpP2 protein was extracted and purified from the *H. parasuis* SC096 strain. The purified OmpP2 protein was extensively dialyzed, and contamination with LPS was revealed by SDS-PAGE gels stained with silver nitrate and further assessed by the Limulus amoebocyte lysate assay. LPS contamination in the porin preparation was estimated to be approximately 0.001% w/w, compared with the standard *H. parasuis* LPS solution [21]. The protein was analysed by 12% SDS-PAGE, visualized by Coomassie blue staining and identified by the MALDI-TOF-TOF method (Additional file 3). The results showed that the purified protein was the *H. parasuis* OmpP2 protein.

### The effects of loops L1–L8 of *H. parasuis* OmpP2 on proinflammatory cytokine expression in PAMs

First, we examined whether OmpP2 and the synthesized peptides corresponding to loops L1–L8 affected the viability of PAMs when stimulated by different concentrations and at various durations. The results showed that under the different conditions, the cell viability was above 95% (Additional file 4), indicating that the concentrations and durations used were not toxic to the cells. Based on these results, PAMs were stimulated with OmpP2 (5 µg/mL and 10 µg/mL), loops L1–L8 peptides or scrambled peptide (130 nmol/mL and 260 nmol/mL). Next, the mRNA expression levels of the proinflammatory cytokines *IL*-*1α*, *IL*-*1β*, *IL*-*6*, *IL*-*8*, *IL*-*17*, *IL*-*23*, *CCL*-*4* and *CCL*-*5* in PAMs were measured after 6 and 12 h by real-time PCR. Gene expression was normalized to the two internal control genes, *GAPDH* and *RPL4*, and the eight relative cytokine expression levels are presented as the fold change relative to the negative control.

**Figure 1:**
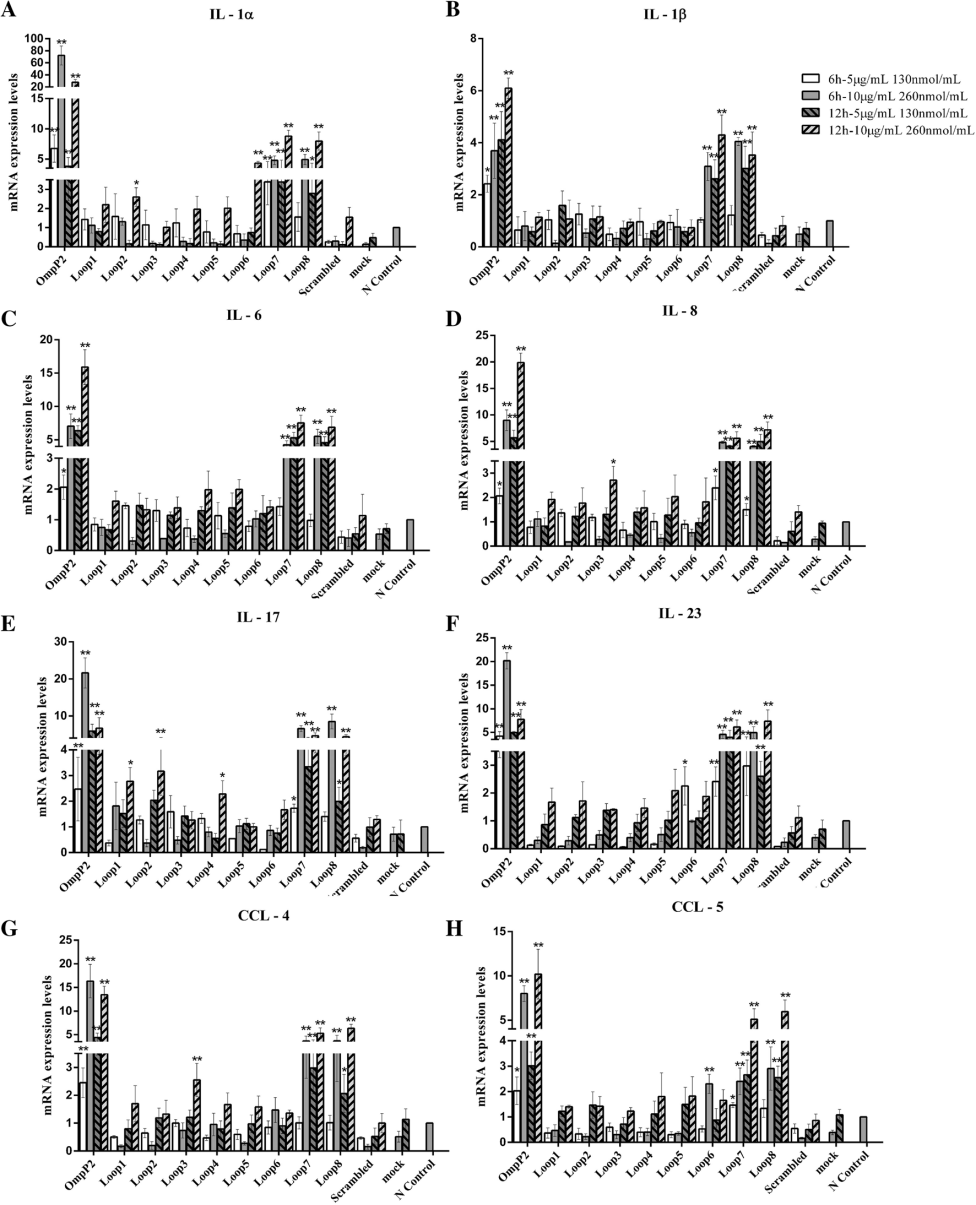
**mRNA expression levels of proinflammatory cytokines in PAMs stimulated by OmpP2 or the eight loop peptides.** PAMs were stimulated with OmpP2 (5 µg/mL and 10 µg/mL), loops L1–L8 or scrambled peptide (130 nmol/mL and 260 nmol/mL) for 6 and 12 h. The mRNA expression levels of *IL*-*1α* (**A**), *IL*-*1β* (**B**), *IL*-*6* (**C**), *IL*-*8* (**D**), *IL*-*17* (**E**), *IL*-*23* (**F**), *CCL*-*4* (**G**) and *CCL*-*5* (**H**) were measured by real-time PCR. The HEPES buffer (pH 7.4) containing 0.25% (w/v) SLS was used as a mock stimulus, and the untreated cells were used as a negative control. Values are presented as the mean ± SD of three independent experiments. Data were analysed using one-way ANOVA. *p < 0.05, **p < 0.01 compared with the negative control.

As shown in Figure 1, *IL*-*1α*, *IL*-*1β*, *IL*-*6* and *IL*-*8* mRNA expression levels were significantly upregulated after PAMs were stimulated with OmpP2 at concentrations of 5 and 10 µg/mL for 6 and 12 h, which is consistent with our previous study [12]. In addition, *IL*-*17*, *IL*-*23*, *CCL*-*4* and *CCL*-*5* mRNA expression levels were significantly upregulated. When cells were stimulated with 10 µg/mL OmpP2 for 6 h, the cytokines *IL*-*17* and *IL*-*23* and the chemokine *CCL*-*4* reached the highest mRNA expression levels (*IL*-*17*, ~21.6-fold over the mock control; *IL*-*23*, ~20.2-fold, and *CCL*-*4*, ~16.32-fold; *p* < 0.01). After a 12-h stimulation, the chemokine *CCL*-*5* reached the highest mRNA expression level (~10.20-fold over the mock control; *p *< 0.01).

Then, we used synthesized linear peptides corresponding to loops L1–L8 of OmpP2 to stimulate PAMs. As shown in Figure 1, the mRNA expression levels of *IL*-*1α*, *IL*-*1β*, *IL*-*6*, *IL*-*8*, *IL*-*17*, *IL*-*23*, *CCL*-*4* and *CCL*-*5* were upregulated by loop peptides compared with the negative control. Among them, both loops L7 and L8 significantly upregulated the mRNA expression levels of the proinflammatory factors in a time- and dose-dependent manner (*p *< 0.05 or *p *< 0.01). In contrast, the scrambled peptide, which had the same composition as loop L7 but in a different order, did not induce significant cytokine expression. These results indicate that both loops L7 and L8 contributed to the OmpP2-induced inflammatory response in PAMs incubated with OmpP2 from *H. parasuis*. Loops L7 and L8 had a similar effect on the mRNA expression levels of proinflammatory factors in PAMs. When the cells were stimulated by loops L7 or L8 at 260 nmol/mL for 12 h, the mRNA expression levels of the proinflammatory factors were the highest (*IL*-*1α*, ~8.0-fold over the negative control; *IL*-*6*, ~7.0-fold; *IL*-*8*, ~5.7-fold; *IL*-*17*, ~6.6-fold; *IL*-*23*, ~6.2-fold; *CCL*-*4*, ~5.3-fold; and *CCL*-*5*, ~5.1-fold; *p *< 0.01). Nevertheless, the highest *IL*-*1β* mRNA expression level was observed upon loop L7 (260 nmol/mL) stimulation for 12 h and loop L8 (260 nmol/mL) stimulation for 6 h (*p *< 0.01).

**Figure 2:**
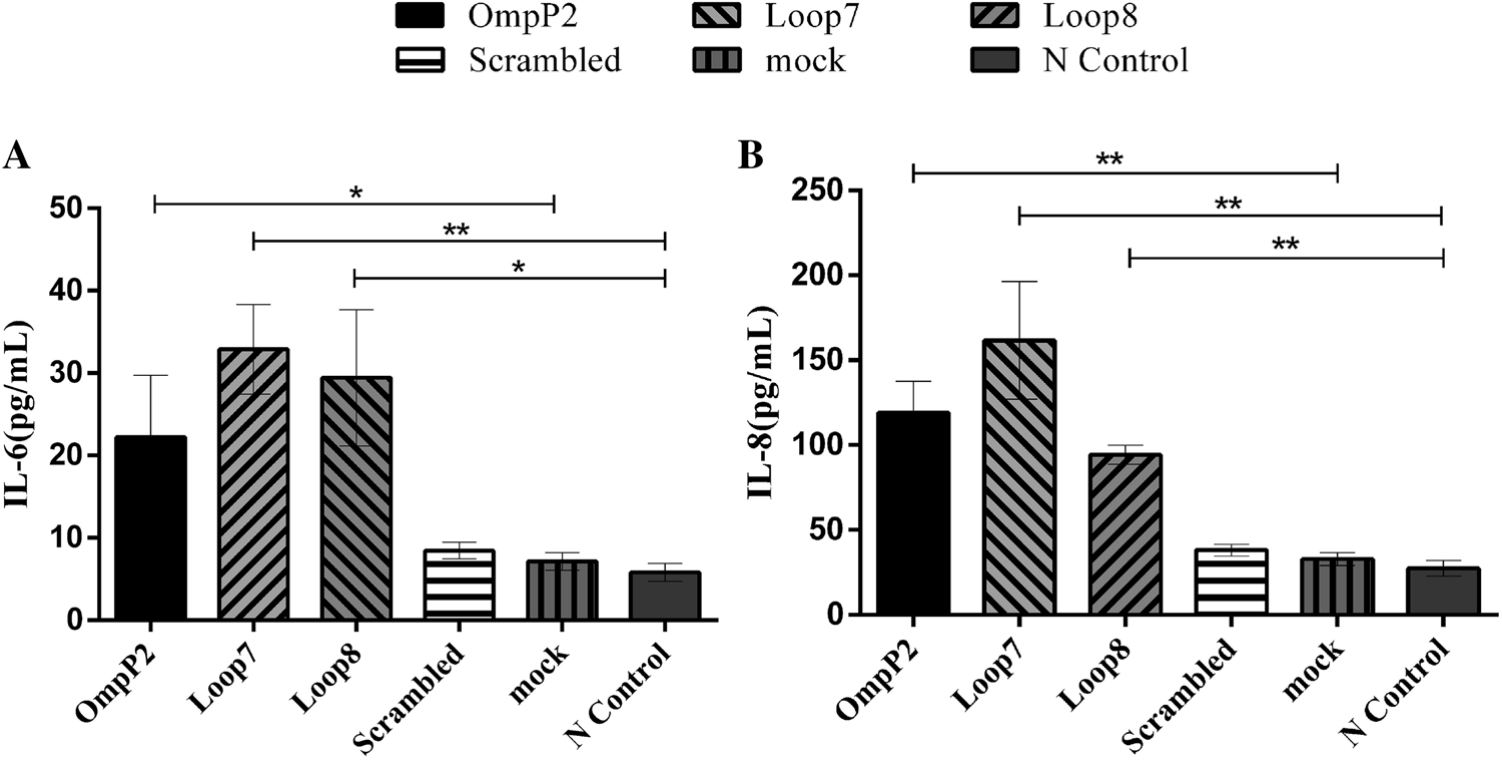
**IL-6 and IL-8 protein release levels by L7 or L8 loop-stimulated PAMs.** PAMs were stimulated with OmpP2 (10 µg/mL), loops L7 and L8 or scrambled peptide (260 nmol/mL) for 12 h. The content of IL-6 (**A**) and IL-8 (**B**) was measured by ELISA. HEPES buffer (pH 7.4) containing 0.25% (w/v) SLS was used as a mock stimulus, and the untreated cells were used as a negative control. Values are presented as the mean ± SD of three independent experiments. Data were analysed using one-way ANOVA. *p < 0.05, **p < 0.01 compared with the negative control.

To assess the effects of both loops L7 and L8 on IL-6 and IL-8 protein release, cell culture supernatants were collected after stimulation with OmpP2 (10 µg/mL), loops L7 and L8 or scrambled peptide (260 nmol/mL) for 12 h. The protein concentration in the supernatants was significantly affected by OmpP2 and by loops L7 and L8 (Figure 2). After OmpP2 stimulation, the protein expression levels of IL-6 and IL-8 (3.1- and 3.6-fold greater than that of the mock control, respectively) were significantly upregulated (*p *< 0.05 and *p *< 0.01, respectively). Furthermore, both loops L7 and L8 significantly induced IL-6 and IL-8 secretion (*p *< 0.01); the IL-6 protein expression level was approximately 5-fold higher than that of the negative control, and the highest expression level of IL-8 protein was observed upon loop L7 stimulation (~5.8-fold higher than that of the negative control). In contrast, after scrambled peptide stimulation, the protein expression levels of IL-6 and IL-8 were not significantly upregulated compared with the negative control (*p *> 0.05). Therefore, the results indicated that both loops L7 and L8 mediated the OmpP2-induced protein expression of IL-6 and IL-8 in PAMs.

### The *ompP2ΔLoop7* and *ompP2ΔLoop8* mutants induce cytokine mRNA expression in PAMs

To further determine the role of loops L7 and L8 in OmpP2-induced cellular inflammatory responses, the *ompP2ΔLoop7* and *ompP2ΔLoop8* mutants of the *H. parasuis* SC096 strain were constructed by natural transformation (Additional file 5). Sequencing analysis of the transformants indicated that the *ompP2ΔLoop7* and *ompP2ΔLoop8* mutants were successfully constructed.

First, we examined whether *ompP2ΔLoop7*- and *ompP2ΔLoop8*-derived OmpP2 affected the viability of PAMs using a CCK-8 kit. We found that under the tested conditions, the cell viability was above 95% (Additional file 4), indicating that these OmpP2 mutants were not toxic to the cells. Next, PAMs were stimulated with *ompP2ΔLoop7*- or *ompP2ΔLoop8*-derived OmpP2 (5 µg/mL and 10 µg/mL), and the mRNA expression of *IL*-*1α*, *IL*-*1β*, *IL*-*6*, *IL*-*8*, *IL*-*17*, *IL*-*23*, *CCL*-*4* and *CCL*-*5* in PAMs was measured via real-time PCR after 6 and 12 h of incubation. Gene expression levels were normalized to two internal control genes (*GAPDH* and *RPL4*), and the relative cytokine expression levels are presented as the fold change relative to the mock control.

**Figure 3:**
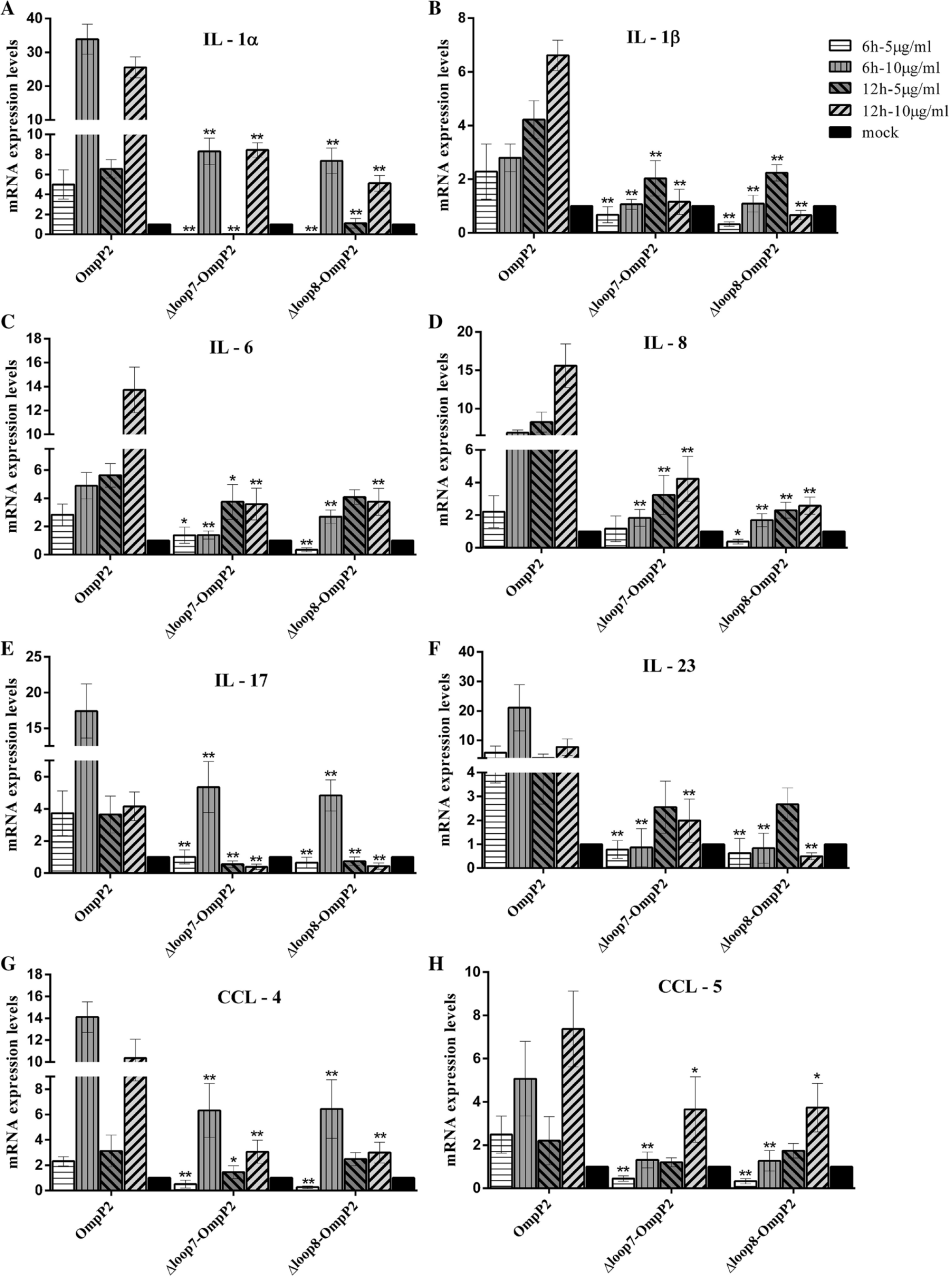
**mRNA expression of proinflammatory cytokines in OmpP2-stimulated PAMs.** PAMs were stimulated with OmpP2 porins, *ompP2ΔLoop7*- or *ompP2ΔLoop8*-derived OmpP2 (5 µg/mL and 10 µg/mL) for 6 and 12 h. The mRNA expression levels of *IL*-*1α* (**A**), *IL*-*1β* (**B**), *IL*-*6* (**C**), *IL*-*8* (**D**), *IL*-*17* (**E**), *IL*-*23* (**F**), *CCL*-*4* (**G**) and *CCL*-*5* (**H**) were measured by real-time PCR. HEPES buffer (pH 7.4) containing 0.25% (w/v) SLS was used as a mock stimulus. Values are presented as the mean ± SD of three independent experiments. Data were analysed using one-way ANOVA. *p < 0.05, **p < 0.01 compared with the mock control.

As shown in Figure 3, the mRNA expression of cytokines and chemokines induced by *ompP2ΔLoop7* and *ompP2ΔLoop8* OmpP2 mutants was noticeably decreased compared with those induced by SC096 OmpP2 in a concentration- or time-dependent manner. When these mutant OmpP2s were applied at 5 µg/mL or 10 µg/mL for 6 h, the mRNA expression levels of the proinflammatory factors were significantly lower (by 50–95%) than those following SC096 OmpP2 stimulation. Similarly, at 12 h, the induction of cytokine and chemokine mRNA expression by *ompP2ΔLoop7* and *ompP2ΔLoop8* OmpP2 was approximately 10–50% of that induced by SC096 OmpP2.

### Amino acid sequence analysis of loops L7 and L8

To elucidate the biological conservation of loops L7 and L8, we compared the amino acid sequences of OmpP2 loops L7 and L8 from 15 serovars of *H. parasuis* reference strains. As shown in Table 2, the loop L7 amino acid sequence was highly conserved among the most virulent reference strains, which included serovars 1, 2, 5, 12, 13, 14 and 15, while the loop L8 amino acid sequence showed high conservation among serovars 5, 12, 13, 14 and 15 reference strains. However, the amino acid sequences of both of these loops have several amino acid mutations in serovars 4, 8 and 10 of the virulent reference strains. Our results suggested that both loops L7 and L8 contribute to the OmpP2-induced inflammatory response in PAMs and belong to a relatively conserved region of OmpP2 in the most virulent *H. parasuis* reference strains.

**Table 2:** **Amino acid sequence of OmpP2 loops L7 and L8 of the 15 serovars of** ***H. parasuis*** **reference strains**

Serovar (strains)	Loop7	Loop8
Clinical isolate strains		
Serovar SC096	GTYKDKAYKATA	NKDSNNKKVTDQA
Serovar 1 (No. 4)	· · · · · · · · · · · ·	· · · · · · N· · · · · ·
Serovar 2 (SW140)	· · · · · · · · · · · ·	· · · · · · · · · · · K·
Serovar 3 (SW114)	· · · · VEDF · V· ·	· · · · D· N· · · · K·
Serovar 4 (SW124)	· · · · VEDF · · · ·	· · N· D· N· · · · K·
Serovar 5 (Nagasaki)	· · · · · · · · · · · ·	· · · · · · · · · · · · ·
Serovar 6 (131)	· · · · VED· · · · ·	· · · · D· N· · · · K·
Serovar 7 (174)	· · · · · · · · · · · ·	· · · · · · · · · · · · ·
Reference strains		
Serovar 8 (C5)	· · · · VEDF ·V · ·	· · · · D· N· · · · K·
Serovar 9 (D74)	· · · · V· D· · · · ·	· · · · D· N· · · · K·
Serovar 10 (H555)	· · · · VEDF ·V · ·	· · · · D· N· · · · K·
Serovar 11 (H465)	· · · · VE ·F · · · ·	· · · · D· N· · · · K·
Serovar 12 (H425)	· · · · · · · · · · · ·	· · · · · · · · · · · · ·
Serovar 13 (84-17975)	· · · · · · · · · · · ·	· · · · · · · · · · · · ·
Serovar 14 (84-22113)	· · · · · · · · · · · ·	· · · · · · · · · · · · ·
Serovar 15 (84-15995)	· · · · · · · · · · · ·	· · · · · · · · · · · · ·

### Loops L7 and L8 induce the NF-κB and MAPK pathways in PAMs

To further investigate which signalling pathways are activated by loops L7 and L8 in the OmpP2-induced inflammatory response, we examined the NF-κB and MAPK signalling pathways by co-transfecting PAMs with the luciferase reporter plasmid pNF-κb-Luc or pAP-1-Luc, respectively, or with pRL-TK, which served as an internal control. At 24 h after transfection, PAMs were treated with OmpP2 (5 µg/mL and 10 µg/mL), loops L7 and L8 or a scrambled peptide (130 nmol/mL and 260 nmol/mL) for 12 h. In addition, the cells were stimulated with TNF-α (40 ng/mL) as a positive control, HEPES buffer (pH 7.4) containing 0.25% (w/v) SLS as a mock stimulus and untreated cells were used as a negative control.

**Figure 4:**
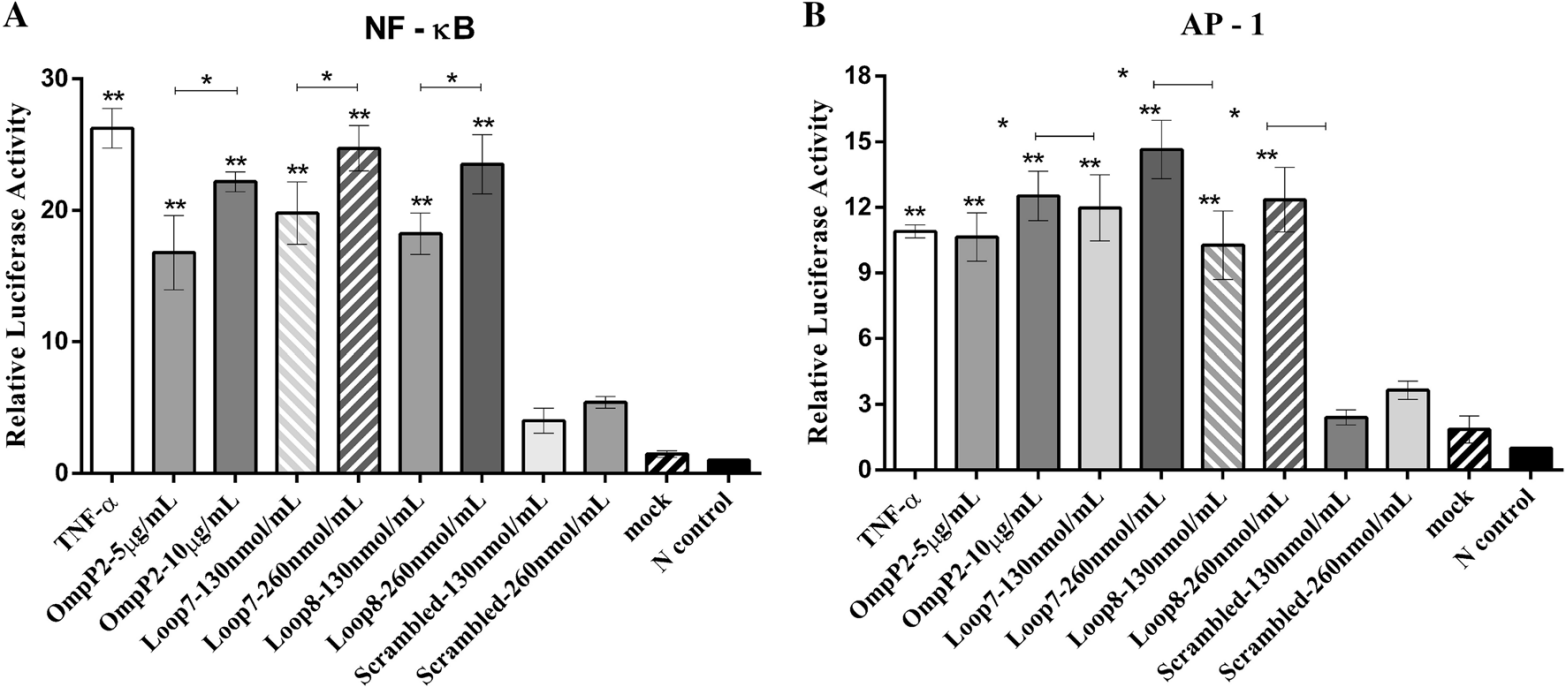
**Loops L7 and L8 activated NF-κB and AP-1 reporters in PAMs.** PAMs were stimulated with OmpP2 (5 µg/mL and 10 µg/mL), loops L7 and L8 or scrambled peptide (130 nmol/mL and 260 nmol/mL, respectively) for 12 h; TNF-α was used as the positive control, HEPES buffer (pH 7.4) containing 0.25% (w/v) SLS as the mock stimulus and untreated cells as the negative control. The dual-luciferase assay activity levels based on luciferase reporter assays of NF-κB and AP-1 are shown. The results are presented as the mean ± SD. Data were analysed using one-way ANOVA. *p < 0.05, **p < 0.01 compared with the negative control.

The results showed that the activity levels based on luciferase reporter assays of NF-κB and AP-1 significantly increased in a dose-dependent manner in the PAMs treated with OmpP2, loop L7, or loop L8 compared with the mock-treated and negative control PAMs (*p *< 0.01; Figure 4). When loops L7 and L8 were used at 260 nmol/mL, they both induced the highest level of NF-κB-mediated fluorescence (~23-fold greater than that in the negative control; *p *< 0.01). The AP-1-mediated fluorescence was significantly increased after loop L7 stimulation at 260 nmol/mL, which was 14.64-fold greater than that in the negative control (*p *< 0.01). In contrast, the activity levels based on luciferase reporter assays of NF-κB and AP-1 were not significantly increased in the PAMs treated with the scrambled peptide compared with the negative control PAMs (*p *> 0.05).

## Discussion

Molecules spanning the outer membrane of gram-negative bacteria are involved in host–cell interactions [1]. Thus, it is important to define the bacterial molecules that react with complementary structures on eukaryotic cells, as this probably represents an important step in the host response to bacterial infection. Previous studies have confirmed that the OmpP2 protein plays a fundamental role in the pathogenesis of gram-negative infections [9, 10, 17, 22]. Moreover, OmpP2 stimulates an immunological response, inducing the release of several cytokines in host cells [22, 23]. In our previous study, OmpP2 purified from virulent *H. parasuis* upregulated the mRNA expression levels of *IL*-*1α*, *IL*-*1β*, *IL*-*6* and *IL*-*8* in PAMs in vitro [12], suggesting that OmpP2 may possess immunomodulatory activity during *H. parasuis* infection.

In this study, we confirmed that the OmpP2 protein purified from the virulent *H. parasuis* SC096 strain upregulated the mRNA expression of the cytokines *IL*-*17* and *IL*-*23* and of the chemokines *CCL*-*4* and *CCL*-*5*. IL-17 (also called IL-17A) of the IL-17 family is a proinflammatory cytokine that can trigger cascades of events that result in neutrophil recruitment, inflammation and host defence [24]. Pathological production of IL-17 leads to excessive inflammation and overt tissue damage [24, 25]. As a heterodimeric protein, IL-23, in conjunction with IL-1, contributes to the expansion and maintenance of Th17 cells that activate the release of the cytokines IL-6, IL-17, IL-22 and TNF-α, all of which can induce inflammatory responses [26–28]. In this study, we demonstrated that *H. parasuis* OmpP2 significantly upregulated the mRNA expression of *IL*-*17* and *IL*-*23*, suggesting that *H. parasuis* infection can induce IL-17 and IL-23 expression in host cells or in pigs. However, the regulatory mechanism of OmpP2-induced IL-17 and IL-23 expression involving inflammatory reactions or host defence by *H. parasuis* infection is unknown. As members of the CC chemokine subfamily, CCL-4 and CCL-5 play important roles in the inflammatory response during a variety of bacterial infections [29]. It has been confirmed that *H. parasuis* infection induced CCL-4 and CCL-5 expression in host cells through the NF-κB and MAPK signalling pathways [4, 5]. In this study, we found that OmpP2 mediated CCL-4 and CCL-5 mRNA expression in host cells infected by *H. parasuis*.

As an important surface component of *H. parasuis*, the OmpP2 protein consists of eight or nine surface-exposed loops of variable length on the external surface of the bacterial membrane [13–15]. Several studies have demonstrated that the biological activities of OmpP2 are linked to different surface-exposed loops. Furthermore, the high degree of surface accessibility of the loop regions, which are responsible for most of the biological activity of porins, is probably an important factor for interacting with target host cells [15, 17]. Data from the literature have indicated that different surface-exposed loops of the OmpP2 protein in gram-negative bacteria play different roles in inflammatory and immunological responses. For instance, loops L5 and L6 of *H. influenza* have been confirmed to be the target of serum bactericidal activity against different strains [30]. Loop L7 of *H. influenza* OmpP2 proteins, which has been shown to be the most active peptide, upregulate proinflammatory cytokines in host cells [17]. Furthermore, loop L7 of *Klebsiella pneumoniae* has been reported to be involved in the interaction with C1q, the first component of the classical pathway of the complement system, and together, they can activate the complement cascade [18]. This study is the first to identify the amino acid sequences of *H. parasuis* OmpP2 that are likely to be involved in the expression of proinflammatory factors. First, we synthesized linear peptides corresponding to loops of L1–L8 of *H. parasuis* OmpP2 to stimulate PAMs. Both loops L7 and L8 significantly upregulated the mRNA expression levels of *IL*-*1α*, *IL*-*1β*, *IL*-*6*, *IL*-*8*, *IL*-*17*, *IL*-*23*, *CCL*-*4* and *CCL*-*5* in a time- and dose-dependent manner. In addition, we constructed *ompP2ΔLoop7* and *ompP2ΔLoop8* mutant strains of *H. parasuis* and isolated the OmpP2 protein to stimulate PAMs. The results exhibited significantly decreased mRNA expression levels of *IL*-*1α*, *IL*-*1β*, *IL*-*6*, *IL*-*8*, *IL*-*17*, *IL*-*23*, *CCL*-*4* and *CCL*-*5*. Therefore, the above results suggest that both loops L7 and L8 are involved in the *H. parasuis* OmpP2-induced proinflammatory response and that they are the most active loops of this protein.

In gram-negative bacteria, the identification of active conserved domains in porins suggests that it may be possible to generate specific inhibitors, thus contributing to the development of potential vaccines and antibacterial drugs [17, 31]. In this study, the sequences of *H. parasuis* OmpP2 from 15 serovar reference strains were aligned with the SC096 strain, and the sequences of loops L7 and L8 are shown in Table 2. The alignment results showed that loops L7 and L8 are relatively conserved in the OmpP2 protein among the most virulent *H. parasuis* reference strains; however, whether both loops L7 and L8 are active peptides in the immune response of *H. parasuis* OmpP2 remains to be further studied. In other bacteria, the loop L7 sequence of the OmpP2 protein can be included among the microbial structures that initiate innate immune responses, and mutations in its amino acids affect the bacterial infection process by affecting OmpP2 [31]. In *H. parasuis*, there are individual amino acid mutations in the sequences of loops L7 and L8 in some of the reference strains; however, whether these mutations affect OmpP2 requires further investigation.

After *H. parasuis* infection, it is recognized by the corresponding receptors (TLR1, TLR2, TLR4, TLR6, and NOD1/2) and regulates the expression of a number of cytokines and chemokines through the NF-κB and/or MAPK signal transduction pathways, thereby causing serious inflammatory reactions [4–6, 32]. It is still unclear whether OmpP2 and specifically loops L7 and L8 play important roles in signalling pathways. In this study, we used the luciferase reporter plasmids pNF-κb-Luc and pAP-1-Luc to investigate the NF-κB and MAPK signalling pathways. The results indicated that OmpP2 via loops L7 and L8 activated the NF-κB and AP-1 luciferase reporters in a dose-dependent manner, suggesting that these loops are involved in the OmpP2-mediated induction of proinflammatory cytokines and chemokines in PAMs, possibly by regulating the NF-κB and MAPK signalling pathways, during *H. parasuis* infection. However, the detailed molecular mechanisms of signalling pathway activation by OmpP2 via loops L7 and L8 still require further investigation.

In summary, this study demonstrated that both loops L7 and L8 of *H. parasuis* OmpP2 had a significant effect on inducing the expression of proinflammatory factors in PAMs, including the cytokines *IL*-*1α*, *IL*-*1β*, *IL*-*6*, *IL*-*8*, *IL*-*17* and *IL*-*23* and the chemokines *CCL*-*4* and *CCL*-*5*. Furthermore, L7 and L8 significantly upregulated the NF-κB and AP-1 activity levels based on luciferase reporter assays. Therefore, our results demonstrated that the surface-exposed loops L7 and L8 of *H. parasuis* OmpP2 induced the expression of proinflammatory cytokines, possibly by activating the NF-κB and MAPK signalling pathways, in cells infected by *H. parasuis*.

## Supplementary information

**Additional file 1. Amino acid sequences of the predicted linear surface-exposed loops of OmpP2 from the virulent*H. parasuis*SC096 strain by PRED-TMBB software.**

**Additional file 2. Sequences of the PCR primers used in this study.**

**Additional file 3. OmpP2 profiles of the** ***H. parasuis*** **SC096 strain.** Lane 1, *H. parasuis* SC096 strain; Lane 2, *ompP2Loop7* mutant; Lane 3, *ompP2Loop8* mutant; Lane M, protein molecular marker standard.

**Additional file 4. Effect of OmpP2, synthetic peptides and** ***ompP2ΔLoop7*- and** ***ompP2ΔLoop8*-derived OmpP2 on viability of PAMs in vitro**. Cell viability was measured by the CCK-8 assay. Data are expressed as the mean ± SD of triplicate samples from at least three independent experiments.

**Additional file 5. Construction and characterization of the** ***ompP2Loop7*** **and** ***ompP2Loop8*** **mutants.** Part 1 shows the map of the *H. parasuis* SC096 strain. Part 2 shows the map of the *ompP2* (Gm^R^) insertion mutant. Part 3 shows the map of the *ompP2Loop7* gene for *H. parasuis* SC096. Part 4 shows the map of the *ompP2Loop8* gene for *H. parasuis* SC096.

## Abbreviations

*H. parasuis*: *Haemophilus* (*Glaesserella*) *parasuis*
*H. influenza*: *Haemophilus influenzae*
OmpP2: outer membrane protein P2
PAMs: porcine alveolar macrophages
*E. coli*: *Escherichia coli*
TSA: trypticase soy agar
TSB: trypticase soy broth
NAD: nicotinamide adenine dinucleotide
SLS: sodium laser sulfate
LPS: lipopolysaccharide
MALDI-TOF-TOF: matrix-assisted laser desorption/ionization time-of-flight
HPLC: high-performance liquid chromatography
CCK-8: cell counting kit-8
GAPDH: glyceraldehyde-3-phosphate dehydrogenase
RPL4: ribosomal protein L-4
CCL-4: C–C motif chemokine ligand 4
TLR1: Toll-like receptor 1
TLR2: Toll-like receptor 2
TLR4: Toll-like receptor 4
TLR6: Toll-like receptor 6
NOD1/2: NOD-like receptor 1/2
MAPK: mitogen-activated protein kinase
SDS-PAGE: sodium dodecyl sulphate-polyacrylamide gel electrophoresis
