# Supplementary material for: Surface-exposed loops L7 and L8 of Haemophilus (Glaesserella) parasuis OmpP2 contribute to the expression of proinflammatory cytokines in porcine alveolar macrophages
Source: Vet Res. 2019 Nov 29;50:105. doi: 10.1186/s13567-019-0721-4 (PMC6884870; doi:10.1186/s13567-019-0721-4)
Supplement: Supplementary file 1 — Additional file 1. Amino acid sequences of the predicted linear surface-exposed loops of OmpP2 from the virulentH. parasuisSC096 strain by PRED-TMBB software. [file 13567_2019_721_MOESM1_ESM.doc]

**Additional file 1. Amino acid sequence of the predicted linear surface-exposed loop peptide of virulent *H. parasuis* SC096 strain OmpP2 by PRED-TMBB software.**

| **Loop** | Amino acid sequence |
| --- | --- |
| Loop 1 | NH2-EEQATKEKGQSSTRGHTNLKNNSSR-COOH |
| Loop 2 | NH2-DSNSENAAGWGDVKT-COOH |
| Loop 3 | NH2-GKQAVIGDSIGQAGFDKVYGVGTGGIKYSANNTNKKGFDILTDSSDSA-COOH |
| Loop 4 | NH2-ANERNNKGEVKVDSAKSG-COOH |
| Loop 5 | NH2-DDYSGSSGSVSFNKKDK-COOH |
| Loop 6 | NH2-LKTDNVKEKIDF-COOH |
| Loop 7 | NH2-GTYKDKAYKATA-COOH |
| Loop 8 | NH2-NKDSNNKKVTDQA-COOH |
| Scrambled | NH2-TAYTKADKKYAG-COOH |
