# Supplementary material for: Surface-exposed loops L7 and L8 of Haemophilus (Glaesserella) parasuis OmpP2 contribute to the expression of proinflammatory cytokines in porcine alveolar macrophages
Source: Vet Res. 2019 Nov 29;50:105. doi: 10.1186/s13567-019-0721-4 (PMC6884870; doi:10.1186/s13567-019-0721-4)
Supplement: Supplementary file 2 — Additional file 2. Sequences of the PCR primers used in this study. [file 13567_2019_721_MOESM2_ESM.doc]

**Additional file 2. The sequence of PCR primers used in this study.**

| Primer | Primer sequence(5'–3') |
| --- | --- |
| **Confirmation of gene expression by real-time RT-PCR** | |
| GAPDH-F  GAPDH-R | CCCCAACGTGTCGGTTGT  CCTGCTCCACCTTCTTGA |
| RPL4-F  RPL4-R | GCTCTATGGCACTTGGCGT  GCGGAGGGCTCTTTGGAT |
| IL-1ɑ-F  IL-1ɑ-R | GAAGAAGAGACGGTTGAG  GCTGTATGTTGCTGA |
| IL-1β-F  IL-1β-R | ACCTGGACCTTGGTTCTCTG  CATCTGCCTGATGCTCTTGT |
| IL-6-F  IL-6-R | AATCCAGACAAAGCCACCC  TCCACTCGTTCTGTGACTGC |
| IL-8-F  IL-8-R | TAGGACCAGAGCCAGGAAGA  AGCAGGAAAACTGCCAAGAA |
| IL-17-F  IL-17-R | CTCTCGTGAAGGCGGGAATC  GTAATCTGAGGGCCGTCTGG |
| IL-23-F  IL-23-R | GGCACAGTGGCCCATAAATC  GCAGCAATTCAGGGTCCAAG |
| CCL-4-F  CCL-4-R | AGCGCTCTCAGCACCAATG  TCCGCACGGTGTATGTGAA |
| CCL-5-F  CCL-5-R | CAGCATCAGCCTCCCCATA  GGGCGGGAGAGGTAGGAAA |
| **Construction of Loop deletion mutants** | |
| P1(P2loop-Km-BamHI-F) | AGACGTGGATCCTTTTATGGACAGCAAGCGAA |
| P2(P2loop-down-SalI-R ) | AGACGTGTCGACGGTCGAACCACGTCAATGTG |
| P3(P2loop-up-EcoRIuss-F) | AGACGTGAATTCACCGCTTGTTTCTTTGACGGAGCCATGAG |
| P4(P2loop-up-BamHI-R) | AGACGTGGATCCTTACCATAATACACGTAAAC |
| P5(P2-lp7-F) | TGTATGGAAACTACTCTTATCATCAATTCATGTTAGGTGC |
| P6(P2-lp7-R) | ATAAGAGTAGTTTCCATACA |
| P7(P2-lp8-F) | TTGAAGGTCGTTTAATCAAGCTTGGTGTTGGTTTACGTGT |
| P8(P2-lp8-R) | CTTGATTAAACGACCTTCAA |
