# Supplementary figures and images for: Surface-exposed loops L7 and L8 of Haemophilus (Glaesserella) parasuis OmpP2 contribute to the expression of proinflammatory cytokines in porcine alveolar macrophages
Source: Vet Res. 2019 Nov 29;50:105. doi: 10.1186/s13567-019-0721-4 (PMC6884870; doi:10.1186/s13567-019-0721-4)

**
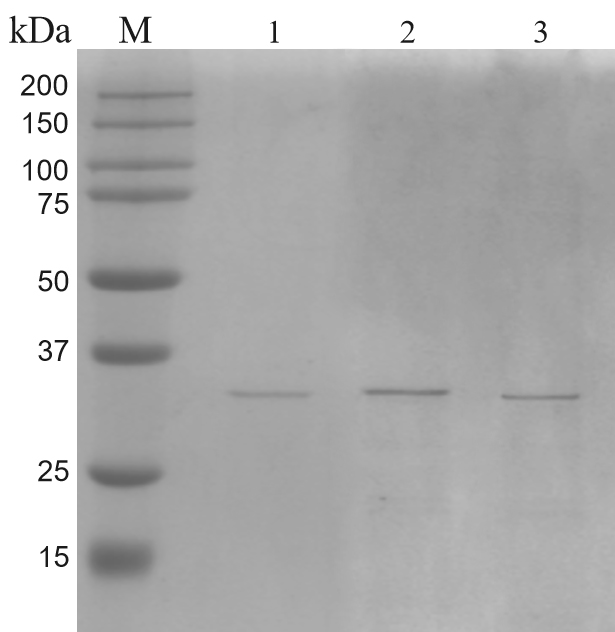
**

Supplement: Supplementary file 3 — Additional file 3. OmpP2 profiles of the H. parasuis SC096 strain. Lane 1, H. parasuis SC096 strain; Lane 2, ompP2Loop7 mutant; Lane 3, ompP2Loop8 mutant; Lane M, protein molecular marker standard. [file 13567_2019_721_MOESM3_ESM.doc]

**
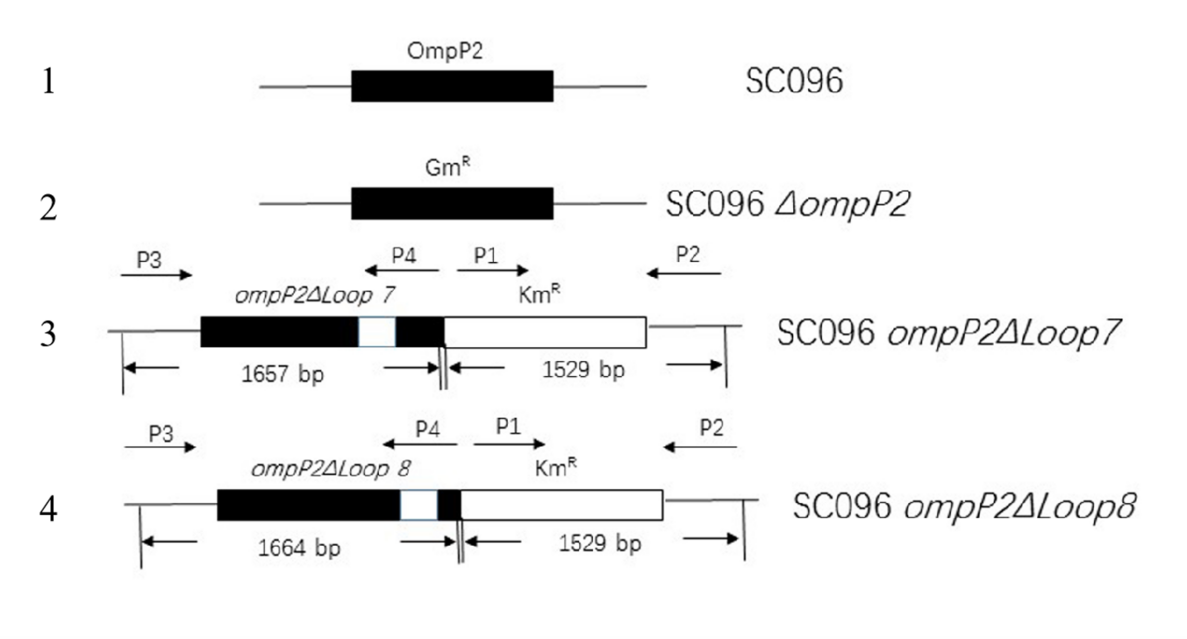
**

Supplement: Supplementary file 5 — Additional file 5. Construction and characterization of the ompP2Loop7 and ompP2Loop8 mutants. Part 1 shows the map of the H. parasuis SC096 strain. Part 2 shows the map of the ompP2 (GmR) insertion mutant. Part 3 shows the map of the ompP2Loop7 gene for H. parasuis SC096. Part 4 shows the map of the ompP2Loop8 gene for H. parasuis SC096. [file 13567_2019_721_MOESM5_ESM.doc]
