# Supplementary material for: Surface-exposed loops L7 and L8 of Haemophilus (Glaesserella) parasuis OmpP2 contribute to the expression of proinflammatory cytokines in porcine alveolar macrophages
Source: Vet Res. 2019 Nov 29;50:105. doi: 10.1186/s13567-019-0721-4 (PMC6884870; doi:10.1186/s13567-019-0721-4)
Supplement: Supplementary file 4 — Additional file 4. Effect of OmpP2, synthetic peptides and ompP2ΔLoop7- and ompP2ΔLoop8-derived OmpP2 on viability of PAMs in vitro. Cell viability was measured by the CCK-8 assay. Data are expressed as the mean ± SD of triplicate samples from at least three independent experiments. [file 13567_2019_721_MOESM4_ESM.doc]

**Additional file 4.** **Effect of OmpP2, synthetic peptides and *ompP2ΔLoop7* and *8*-derived OmpP2 on PAMs viability in vitro.** Cell viability was measured by the CCK-8 assay. Data are expressed as mean ± SD of triplicate samples from at least three independent experiments.

| Cell viability (%) | | |
| --- | --- | --- |
| Stimulant (concentrations) | Times | |
|  | 6 h | 12 h |
| OmpP2 (5 μg/mL) | 113.78 ± 2.8 | 115.57±0.22 |
| OmpP2 (10 μg/mL) | 102.31 ±3.09 | 121.40±0.48 |
| *ompP2ΔLoop7*-OmpP2 (5 μg/mL) | 105.73 ±5.07 | 126.12±3.13 |
| *ompP2ΔLoop7*-OmpP2 (10 μg/mL) | 107.18 ±7.05 | 133.93±1.17 |
| *ompP2ΔLoop8*-OmpP2 (5 μg/mL) | 107.60 ±6.11 | 131.05 ± 3.46 |
| *ompP2ΔLoop8*-OmpP2 (10 μg/mL) | 101.45 ±7.06 | 123.80 ± 3.11 |
| Loop1 (130 nmol/mL) | 103.73 ± 4.82 | 112.02 ± 1.26 |
| Loop1 (260 nmol/mL) | 95.11 ± 4.31 | 104.61 ± 0.13 |
| Loop2 (130 nmol/mL) | 96.05 ± 5.94 | 108.00 ± 0.42 |
| Loop2 (260 nmol/mL) | 103.53 ± 5.62 | 106.24 ± 1.01 |
| Loop3 (130 nmol/mL) | 115.95 ± 1.99 | 122.12 ± 0.16 |
| Loop3 (260 nmol/mL) | 123.30 ± 1.12 | 124.78 ± 0.16 |
| Loop4 (130 nmol/mL) | 121.51 ± 2.34 | 112.14 ± 0.97 |
| Loop4 (260 nmol/mL) | 125.30 ± 2.86 | 114.30 ± 0.68 |
| Loop5 (130 nmol/mL) | 120.95 ± 2.51 | 116.99 ± 0.38 |
| Loop5 (260 nmol/mL) | 139.83 ± 4.78 | 104.53 ± 1.26 |
| Loop6 (130 nmol/mL) | 115.56 ± 4.57 | 123.92 ± 0.35 |
| Loop6 (260 nmol/mL) | 125.55 ± 2.48 | 126.73 ± 0.52 |
| Loop7 (130 nmol/mL) | 115.01 ± 3.13 | 126.12 ± 0.66 |
| Loop7 (260 nmol/mL) | 122.05 ± 1.11 | 123.09 ± 1.94 |
| Loop8 (130 nmol/mL) | 122.10 ± 1.94 | 113.56 ± 0.29 |
| Loop8 (260 nmol/mL) | 119.90 ± 3.81 | 103.99 ± 1.18 |
